# The Impacts and Economic Analysis of Jack Mackerel Meal Inclusion in Low Fish Meal Diets on the Growth and Feed Availability of Juvenile Rockfish (*Sebastes schlegeli*)

**DOI:** 10.3390/ani15010062

**Published:** 2024-12-30

**Authors:** Yu Jin Sim, June Kim, Sung Hwoan Cho

**Affiliations:** 1 Department of Convergence Interdisciplinary Education of Maritime & Ocean Contents, Korea Maritime and Ocean University, Busan 49112, Republic of Korea; dbfl1543@naver.com (Y.J.S.); rlawns6766@naver.com (J.K.); 2 Division of Convergence on Marine Science, Korea Maritime and Ocean University, Busan 49112, Republic of Korea

**Keywords:** animal by-product meals, fish meal, substitution source, substitution level, fatty acid, amino acid

## Abstract

A two-way {dietary substitution source [chicken by-product meal (CBM), meat meal (MM), and tuna by-product meal (TBM)] × substitution level (25% and 50%)} ANOVA experimental design was adopted. The control (Con) diet included 55% FM. In the Con diet, 25% and 50% of FM were substituted with CBM, MM, and TBM, with the inclusion of 22% JMM exhibiting strong attractiveness for rockfish, named CBM25J, CBM50J, MM25J, MM50J, TBM25J, and TBM50J diets, respectively. All experimental diets were assigned to triplicate groups of fish. A total of 525 juvenile fish were distributed into 21 flow-through tanks. The CBM- and TBM-substituted diets achieved greater weight gain and specific growth rates (SGRs) of rockfish than the MM-substituted diets. Moreover, dietary 25% FM substitutions achieved greater weight gain and SGRs of rockfish than dietary 50% FM substitutions. Weight gain and SGRs of rockfish fed the Con, CBM25J, and TBM25J diets were superior to those of rockfish fed the CBM50J, MM50J, and TBM50J diets. Dietary 25% FM substitutions exhibited higher rockfish feed consumption than dietary 50% FM substitutions. None of the proximate compositions, amino and fatty acid profiles, or blood chemistry of the fish were significantly different among dietary treatments. The highest EPI was observed in the CBM25J diet. Conclusively, a 25% FM substitution with CBM, MM, and TBM with the inclusion of 22% JMM in the 55% FM-based diet could be made without reducing the growth and feed consumption of rockfish. Finally, the CBM25J diet produced the highest weight gain and SGRs of rockfish, as well as the highest economic return to farmers.

## Simple Summary

Low fish meal (FM) diets frequently lead to deteriorated palatability, reduced feed consumption by fish, and, eventually, lowered growth performance. To resolve these issues, the inclusion of feed ingredients exhibiting strong attractiveness to a target fish in low FM diets is one of the best options. This study aimed to evaluate the manipulation effect of jack mackerel meal (JMM), which exhibits strong attractiveness to rockfish (*S. schlegeli*), in low FM diets, replacing 25% and 50% FM with various animal proteins [chicken by-product meal (CBM), meat meal (MM), and tuna by-product meal (TBM)], on the growth and feed availability of rockfish; economic analysis was also performed. This experiment revealed that the substitution of CBM, MM, and TBM for 25% FM in diets with 22% JMM inclusion led to comparable or higher weight gain, specific growth rates (SGRs), feed consumption, and economic profit indexes (EPIs) compared to the 55% FM-based diet. This study is very helpful for feed nutritionists/producers in formulating low FM diets for sustainable rockfish culture.

## 1. Introduction

Rockfish (*Sebastes schlegeli*) is an economically important farmed species in the Republic of Korea (*hereafter*, Korea), comprising approximately 18% (14,418 metric tons) of the total annual production (79,651 metric tons) of marine fish in 2023, highlighting its importance to Korea’s fish farming industry [1]. For commercially farmed carnivorous fish species, such as rockfish and olive flounders (*Paralichthys olivaceus*), which require high-protein diets, fish meal (FM) is primarily included as the protein source in commercial feeds due to its rich content of essential amino acids (EAAs) and high palatability over other protein sources [2,3]. However, FM is an unsustainable and expensive ingredient with unstable supply because of its increased demand associated with the ever-increasing global expansion of aquaculture [4]. Therefore, feed nutritionists are looking for a cost-effective replacement that can be constantly supplied without causing nutrient imbalances. Animal and fishery by-products have been widely used as FM alternatives in fish feed globally due to their balanced AA profiles, high levels of digestible protein, and high energy content [5,6,7].

Chicken by-product meal (CBM), a dried, ground, and rendered product from clean parts of chicken carcasses, has been successfully substituted for 50% FM in the diets of olive flounders without causing any reduction in weight gain [8]. In addition, 25% and 28.6% FM was successfully replaced with chicken offal meal [9] and chicken waste meal [10] in 54% and 35% FM-based diets of silver seabream (*Rhabdosargus sarba*) and Asian seabass (*Lates calcarifer*), respectively, without deteriorating growth performance. Therefore, CBM can also be used as a substitute for FM in rockfish feed.

Meat meal (MM) produced from terrestrial animals has been widely used as an attractive alternative for FM in several farmed fish species [11,12,13]. Porcine MM could substitute FM by up to 30% in a 30% FM-based diet without deteriorating the growth of juvenile golden pompanos (*Trachinotus ovatus*) [14]. In addition, up to 60% and 40% FM could be replaceable by pet-grade (over 80% crude protein) MM with supplementation with EAAs (lysine, methionine, and tryptophan) or without supplementation with EAAs in 80 and 65% FM-basal diets of juvenile olive flounders, respectively [12,15].

Tuna by-product meal (TBM) obtained from the residual products of the tuna-canning process [16] has been used as a novel FM replacer in several fish species, including rockfish [17], the spotted rose snapper (*Lutjanus guttatus*) [18], and the Nile tilapia (*Oreochromis niloticus*) [19]. Furthermore, replacements of up to 10.7–12.5% FM with fermented TBM with garlic husks have been made in the feed of juvenile olive flounders without compromising growth [20]. Uyan et al. [21] also proved that dietary replacement of up to 50% FM with tuna muscle by-product meal powder improved the growth of olive flounders and reduced phosphorous discharge to the surroundings.

Despite their potential as substitutes for FM, these alternative sources are inferior to FM in fish feeds because of deteriorated palatability, low digestibility, and imbalanced EAAs [6,7,22]. To address these issues, the application of feed stimulants and enhancers in fish feeds could promote ingestion and the continuation of feeding and lead to enhanced growth rates and the feed consumption of GIFT tilapia (*Oreochromis* sp.) [23], red sea bream (*Pagrus major*) [24], and striped bass (*Morone saxatilis*) [25]. Inosine monophosphate (IMP) and lactic acid in the extracts of jack mackerel (*Trachurus japonicus*) muscle were profoundly responsible for the stimulatory responses in young yellowtails (*Seriola quinqueradiata*) [26]. In our previous studies, JMM exhibited the strongest attractiveness to juvenile rockfish among 16 protein feed ingredients [27]; the optimum inclusion level of JMM was estimated to be 22% in the 55% FM-based diet of rockfish in order to achieve the greatest growth performance and highest feed intake [28]. Furthermore, JMM also acted as a strong feed stimulant in yellowtail feed [29].

To address the challenges of rockfish aquaculture in Korea, the inclusion of JMM in low FM diets for rockfish can significantly improve the substitutability of animal-originated alternative proteins for FM, contributing to the sustainability of the aquaculture industry. However, since JMM (USD 2.61/kg, USD 1 = 1300 KRW) is one of the most expensive FM sources in formulating fish feeds, its economic value should be considered when included in the low FM feed. This experiment, therefore, is designed to perform an economic analysis as well as evaluate the effect of JMM in low FM diets, substituting different levels of FM with diverse animal protein sources on the growth, feed utilization, biochemical composition, and blood chemistry of juvenile rockfish.

## 2. Materials and Methods

### 2.1. Rearing Conditions of the Feeding Trial

Juvenile rockfish were purchased from a private hatchery (Buan-gun, Jeollabuk-do, Republic of Korea). Prior to the start of the experiment, fish were acclimated to the experimental conditions for 2 weeks by providing them with a commercial extruded pellet containing 50% crude protein and 8% crude lipid (Suhyup Feed, Uiryeong-gun, Gyeongsangnam-do, Republic of Korea). A total of 525 juvenile rockfish of uniform size (initial weight of 8.3 g) were randomly distributed into 21 tanks of 50 L flow-through tanks, with 25 fish per tank. Each tank was filled with a 1:1 blend of underground and sand-filtered seawater, with a flow rate of 4.5 L/min. Aeration was continuously supplied to each tank. Water quality was measured daily after the first morning feeding using a digital multimeter (AZ-86031; AZ Instrument Corp., Taichung, Taiwan). Throughout the 8-week feeding period, water temperature ranged from 20.7 °C to 24.1 °C (22.3 ± 0.68 °C; mean ± SD), salinity from 31.2 to 33.6 g/L (32.5 ± 0.58 g/L), pH from pH 7.1 to pH 7.5 (pH 7.3 ± 0.07), and dissolved oxygen from 7.2 to 7.5 mg/L (7.4 ± 0.09 mg/L). Fish were carefully hand-fed to apparent satiation twice daily (08:00 and 17:00) for 8 weeks. The tank bottoms were siphon-cleaned daily, and any dead fish were removed immediately upon observation.

### 2.2. Experimental Design of the Feeding Trial

JMM, CBM, MM, and TBM were purchased from local distributors, Daekyung Oil & Transportation Co., Ltd. (Busan Metropolitan City, Republic of Korea), Hwasong Ind. Co., Ltd. (Seogwipo-si, Jeju Special Self-Governing Province, Republic of Korea), Woosin Food Co., Ltd. (Pocheon-si, Gyonggi-do, Republic of Korea), and Woojinfeed Ind. Co., Ltd. (Incheon Metropolitan City, Republic of Korea), respectively. This study employed a two-way ANOVA experimental design to assess the substitution effect of different levels (25% and 50%) of FM with various animal protein sources (CBM, MM, and TBM) in diets that included 22% JMM, which is the optimum level for rockfish growth performance [28]. The two main effects of the two-way ANOVA were the FM substitution source (CBM, MM, and TBM) and the FM substitution level (25% and 50%) in the rockfish diet. Fifty-five percent FM and 12% fermented soybean meal were used as the protein sources in the control (Con) diet (Table 1). The Con diet also included 21.5% wheat flour and 4.5% each of fish and soybean oils as the carbohydrate and lipid sources, respectively. In the Con diet, 25% and 50% of FM were substituted by CBM, MM, and TBM, along with an additional inclusion of 22% JMM at the expense of FM. These diets are referred to as the CBM25J, CBM50J, MM25J, MM50J, TBM25J, and TBM50J diets, respectively.

Seven experimental diets, each isonitrogenous at 51.5% and isolipidic at 14.5%, were formulated to meet the nutrient requirements of rockfish [30,31]. The ingredients of each diet were thoroughly blended with water at a 3:1 ratio. The mixture was pelletized using a laboratory pellet extruder (SMC-32; SL Company, Incheon Metropolitan City, Republic of Korea). Then, the pelletized feeds were dried at 45 °C in electronic dry machine (SI-2400; SIN IL Drying Machine Co., Ltd., Daegu, Republic of Korea) for 24 h, and stored in refrigerator at –20 °C until use. All experimental diets were assigned to triplicate groups of fish. The total amount of feed supplied to each tank was measured daily; however, uneaten feed was not collected and was included in the total amount of feed supplied.

**Table 1:** Ingredient and chemical composition of the experimental diets (%, DM basis).

	Experimental Diets						
	Con	CBM25J	CBM50J	MM25J	MM50J	TBM25J	TBM50J
Ingredients (%, DM basis)							
Fish meal (FM) ^a^	55.0	19.3	5.5	19.3	5.5	19.3	5.5
Jack mackerel meal (JMM) ^b^		22.0	22.0	22.0	22.0	22.0	22.0
Chicken by-product meal (CBM) ^c^		16.5	33.0				
Meat meal (MM) ^d^				11.8	23.6		
Tuna by-product meal (TBM) ^e^						17.1	34.2
Fermented soybean meal ^f^	12.0	12.0	12.0	12.0	12.0	12.0	12.0
Wheat flour	21.5	19.3	16.6	24.4	27	19.8	17.6
Fish oil	4.5	4.5	4.5	4.5	4.5	4.5	4.5
Soybean oil	4.5	3.9	3.9	3.5	2.9	2.8	1.7
Vitamin premix ^g^	1.0	1.0	1.0	1.0	1.0	1.0	1.0
Mineral premix ^h^	1.0	1.0	1.0	1.0	1.0	1.0	1.0
Choline	0.5	0.5	0.5	0.5	0.5	0.5	0.5
Nutrients (%, DM basis)							
Dry matter	95.0	93.5	91.8	94.2	91.5	93.2	93.9
Crude protein	51.7	51.7	51.8	51.6	51.3	51.4	51.1
Crude lipid	14.3	14.6	14.5	14.4	14.3	14.5	14.5
Ash	10.4	11.1	11.6	9.4	7.7	11.9	12.8
Carbohydrates ^i^	23.6	22.6	22.1	24.6	26.7	22.2	21.6
Gross energy (kcal/g) ^j^	4.3	4.3	4.3	4.3	4.4	4.2	4.2

Con: 55% fish meal (FM)-based diet; CBM25J: dietary 25% FM replacement with chicken by-product (CBM) meal with 22% jack mackerel meal (JMM) inclusion; CBM50J: dietary 50% FM replacement with CBM with 22% JMM inclusion; MM25J: dietary 25% FM replacement with meat meal (MM) with 22% JMM inclusion; MM50J: dietary 50% FM replacement with MM with 22% JMM inclusion; TBM25J: dietary 25% FM replacement with tuna by-product (TBM) with 22% JMM inclusion; TBM50J: dietary 50% FM replacement with TBM with 22% JMM inclusion. ^a^ Fish meal (FM) (crude protein: 74.1%, crude lipid: 7.6%, ash: 14.0%) was imported from Chile [USD 2.00/kg of FM, USD 1 = 1300 KRW (Korean currency)]. ^b^ Jack mackerel meal (JMM) (crude protein: 72.8%, crude lipid: 9.8%, ash: 15.4%) was purchased from Daekyung Oil & Transportation Co., Ltd. (Busan Metropolitan City, Republic of Korea) (USD 2.61/kg of JMM). ^c^ Chicken by-product meal (CBM) (crude protein: 65.0%, crude lipid: 13.6%, ash: 16.4%) (USD 0.72/kg of CBM), ^d^ meat meal (MM) (crude protein: 85.6%, crude lipid: 8.5%, ash: 5.9%) (USD 0.92/kg of MM), and ^e^ tuna by-product meal (TBM) (crude protein: 63.5%, crude lipid: 9.8%, ash: 18.6%) (USD 1.07/kg of TBM) were purchased from Hwasong Ind. Co., Ltd. (Seogwipo-si, Jeju Special Self-Governing Province, Republic of Korea), Woosin Food Co., Ltd. (Pocheon-si, Gyonggi-do, Republic of Korea), and Woojinfeed Ind. Co., Ltd. (Incheon Metropolitan City, Republic of Korea), respectively. ^f^ Fermented soybean meal (crude protein: 55.4%, crude lipid, 1.5%, ash: 6.7%) was purchased from CJ CheilJedang (Seoul, Republic of Korea). ^g^ Vitamin premix (g/kg mix): L-ascorbic acid, 121.2; DL-α-tocopheryl acetate, 18.8; thiamin hydrochloride, 2.7; riboflavin, 9.1; pyridoxine hydrochloride, 1.8; niacin, 36.4; Ca-D-pantothenate, 12.7; myo-inositol, 181.8; D-biotin, 0.27; folic acid, 0.68; p-aminobenzoic acid, 18.2; menadione, 1.8; retinyl acetate, 0.73; cholecalciferol, 0.003; cyanocobalamin, 0.003. ^h^ Mineral premix (g/kg mix): MgSO_4_·7H_2_O, 80.0; NaH_2_PO_4_·2H_2_O, 370.0; KCl, 130.0; ferric citrate, 40.0; ZnSO_4_·7H_2_O, 20.0; Ca-lactate, 356.5; CuCl, 0.2; AlCl_3_·6H_2_O, 0.15; KI, 0.15; Na_2_Se_2_O_3_, 0.01; MnSO_4_·H_2_O, 2.0; CoCl_2_·6H_2_O, 1.0. ^i^ Carbohydrates were calculated by difference [carbohydrate = 100 − (crude protein + crude lipid + ash)]. ^j^ Gross energy (GE) (kcal/g) was calculated at 4 kcal/g for crude proteins and carbohydrates and 9 kcal/g for lipids [32].

### 2.3. Measurement of the Biological Indices of Rockfish

At the end of the 8-week experiment, all live fish were starved for 24 h. For the sampling procedure, the fish were anesthetized with tricaine methanesulfonate (MS-222; MilliporeSigma, Burlington, MA, USA) at a concentration of 100 mg/L. The number and collective weight of the fish were then measured to determine survival and weight gain. Ten fish from each tank were dissected to calculate the biological indices. The performance and biological indices of the rockfish were calculated as follows: Specific growth rate (SGR, %/day) = [Ln final weight of fish (g) − Ln initial weight of fish (g)] × 100/days of the feeding trial (56 days); feed efficiency (FE) = weight gain of fish (g)/total feed consumption (g); protein efficiency ratio (PER) = weight gain of fish (g)/protein consumption (g); protein retention (PR, %) = protein gain of fish (g) × 100/protein consumption (g); condition factor (CF) = body weight of fish (g) × 100/total length of fish (cm)^3^; viscerosomatic index (VSI, %) = viscera weight of fish (g) × 100/body weight of fish (g); and hepatosomatic index (HSI, %) = liver weight of fish (g) × 100/body weight of fish (g).

### 2.4. Biochemical Composition Analysis of the Experimental Diets and Whole-Body Rockfish

Ten fish before the feeding trial and all remaining fish (≥7) from each tank at the end of the 8-week feeding experiment were homogenized to determine the biochemical composition of the whole-body rockfish. The fish were hand-minced for homogenization and stored at –20 °C for further analysis. The chemical composition of the samples was measured according to standard procedures [33]. The Kjeldahl technique (Kjeltec 2100 Distillation Unit, Foss Tecator, Hoganas, Sweden) was used to estimate the crude protein, and an ether-extraction method (Soxtec TM 2043 Fat Extraction System, Foss Tecator, Hoganas, Sweden) was used for crude lipid content estimation. To evaluate moisture content, the samples were dried in an oven at 105 °C for 6 h for dry samples and 24 for wet samples. The ash content was assessed by incinerating the samples in a muffle furnace at 550 °C for 4 h.

The AA profiles of the experimental diets and the whole-body rockfish were examined using an AA analyzer (L-8800 Autoanalyzer: Hitachi, Tokyo, Japan), except for methionine, cysteine, and tryptophan. This analysis was conducted after the fish were hydrolyzed with 6 N HCl at 110 °C for 24 h. Samples were oxidized with performic acid for 24 h at a temperature below 5 °C to produce methionine sulfone and cysteic acid for methionine and cysteine measurement. They were then freeze-dried, hydrolyzed, and analyzed using the same process as for the other AA. High-performance liquid chromatography was used to assess tryptophan concentration (S1125 HPLC pump system, Sykam, Germany).

Fatty acids (FAs) were identified by comparing the whole-body fish and experimental diets to a known standard (37-component FAME mix; Supelco™, St. Louis, MO, USA). For the FA analyses, lipids in the experimental diets and whole-body fish were separated by mixing chloroform and methanol (2:1 *v*/*v*) according to Folch et al. [34]’s study. The FA methyl esters were prepared via transesterification with 14% BF_3_-MeOH (Sigma, St. Louis, MO, USA) and analyzed using gas chromatography (Truce GC; Thermo, Waltham, MA, USA) equipped with a flame ionization detector and an SP^TM^-2560 capillary column (100 × 0.25 mm inner diameter, 0.20 µm film thickness; Supelco, Bellefonte, PA, USA).

### 2.5. Plasma and Serum Analysis of Rockfish

Prior to blood collection, fish were anesthetized with 100 mg/L of MS-222 following the 8-week feeding trial. Blood was drawn from the caudal veins of three anesthetized fish from each tank using a heparinized syringe. The blood from three fish was pooled to create a single sample for each tank. Plasma was extracted via centrifugation of the blood samples at 2710× *g* at 4 °C for 10 min, then stored at −70 °C until analysis. An automatic chemistry system (Fuji Dri-Chem NX500i, Fujifilm, Tokyo, Japan) was used to analyze aspartate aminotransferase (AST), alanine aminotransferase (ALT), alkaline phosphatase (ALP), total bilirubin (T-BIL), total cholesterol (T-CHO), triglyceride (TG), total protein (TP), and albumin (ALB) from the plasma, which was stored at −70 °C.

Prior to blood collection, fish were anesthetized with 100 mg/L MS-222 following the 8-week feeding trial. Blood was drawn from caudal veins of three anesthetized fish from each tank using a syringe. The blood from three fish was pooled to create a single sample for each tank. Serum was extracted via centrifugation of the blood samples at 2710× *g* at 4 °C for 10 min, and red at −70 °C until analysis. Lysozyme activity was measured using a turbidimetric assay according to Lange et al. [35]’s study. In summary, 100 µL of serum was added to a 1.9 mL suspension of *Micrococcus lysodeikticus* (0.2 mg/mL; Sigma, St. Louis, MO, USA) in 0.05 M of sodium phosphate buffer at pH 6.2. The reaction was conducted at 25 °C, and absorbance at 530 nm was measured using a microplate reader (Infinite^®^ 200 PRO; TECAN, Männedorf, canton of Zürich, Switzerland) before and after the 30 min reaction period. Lysozyme activity was defined as the amount of enzyme required to lysate the Gram-positive *M. lysodeikticus* bacterium.

Superoxide dismutase (SOD) was measured using a SOD ELISA kit (MBS705758; MyBiosource, Inc., San Diego, CA, USA). This assay uses the competitive inhibition enzyme immunoassay technique. After adding the substrate solution, the color intensity developed was inversely proportional to the SOD concentration in the sample. Absorbance was measured at 450 nm using a microplate reader, and the concentration was determined by constructing a standard curve.

### 2.6. Economic Analysis of the Study

The economic evaluation of the experimental diets for rockfish was conducted using formulas from the study by Martínez-Llorens et al. [36]: economic conversion ratio (ECR, USD/kg) = feed consumption of fish (kg/fish)/weight gain (kg/fish) × diet price (USD/kg) and economic profit index (EPI, USD/fish) = [final weight (kg/fish) × fish sale price (USD/kg)] − [ECR (USD/kg) × weight gain (kg/fish)].

The price of the experimental diets was calculated by multiplying the ingredient composition of each experimental diet and their price based on the market price. Each ingredient price was as follows: USD 2.00/kg of FM, USD 2.61/kg of JMM, USD 0.72/kg of CBM, USD 0.92/kg of MM, USD 1.07/kg of TBM, USD 0.66/kg of fermented soybean meal, USD 0.52/kg of wheat flour, USD 2.61/kg of fish oil, USD 1.69/kg of soybean oil, USD 7.84/kg of vitamin premix, USD 6.30/kg of mineral premix, and USD 1.39/kg of choline. Juvenile rockfish price was calculated at USD 33.3/kg of fish (1 USD per 30 g of fish).

### 2.7. Statistical Analysis

After testing for normality using Shapiro-Wilk’s test and homogeneity of variances using Levene’s test in SPSS version 24.0 (SPSS Inc., Chicago, IL, USA), a two-way ANOVA was conducted to determine the significance of the main effects, followed by Duncan’s multiple range test [37] to assess the significance of dietary treatment effects. Percentage data were arcsine-transformed before statistical analysis. A p-value of less than 0.05 was considered to indicate a significant difference. The FA profiles of the whole-body rockfish were compared using principal component analysis (PCA) in SPSS version 24.0.

## 3. Results

### 3.1. Amino Acid and Fatty Acid Profiles of the Experimental Diets

All EAAs in JMM were relatively higher than those in FM (Table 2). Among EAAs, in particular, arginine and tryptophan, arginine, histidine, and tryptophan in CBM, MM, and TBM, respectively, were relatively higher than those in FM. An elevated substitution level of FM by CBM, MM, and TBM in diets with the inclusion of JMM led to a decrease in most EAAs. An increased FM replacement level by CBM, MM, and TBM led to an increase in arginine, leucine, and tryptophan in the CBM-substituted diets; arginine in the MM-substituted diets; and arginine, histidine, isoleucine, phenylalanine, and tryptophan in the TBM-substituted diets.

Total monounsaturated FA (∑MUFA), n-3 highly unsaturated FA (∑n-3 HUFA), and n-3 FA (∑n-3), and ∑n-3/total n-6 FA (∑n-6) in JMM were relatively higher than those in FM (Table 3). The total saturated FA (∑SFA) and ∑MUFA in CBM, MM, and TBM were relatively higher than those in FM but lower for ∑n-3 HUFA, ∑n-3, ∑n-6, and ∑n-3/∑n-6. In particular, eicosapentaenoic acid (EPA, 20:5n-3) and docosahexaenoic acid (DHA, 22:6n-3) in FM were relatively higher than those in CBM, MM, and TBM. Increased FM substitution levels by CBM, MM, and TBM in diets led to an increase in ∑SFA and ∑MUFA but a decrease in ∑n-3 HUFA, ∑n-3, and ∑n-3/∑n-6.

### 3.2. Survival and Growth Performance of Rockfish

Survival of rockfish ranged from 96 to 100%, and it was not significantly (*p* > 0.05) influenced by either dietary FM substitution source or substitution level (Table 4).

**Figure 1:**
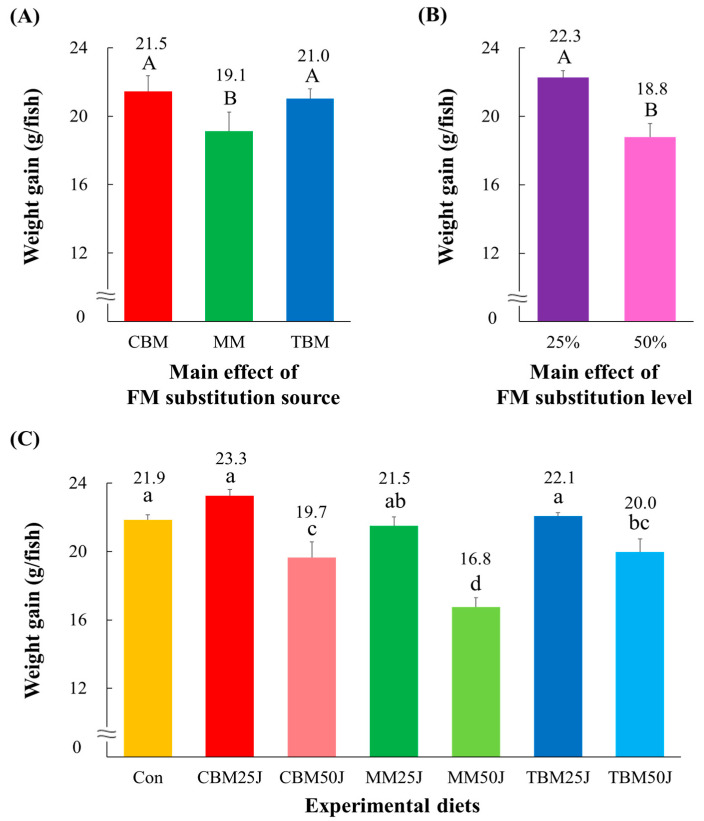
Weight gain (g/fish) of rockfish (*Sebastes schlegeli*) fed the experimental diets for 8 weeks (mean of triplicate ± SE) [(**A**); the main effect of FM substitution source, *p* < 0.005, (**B**); the main effect of FM substitution level, *p* < 0.0001, and (**C**); Duncan’s multiple range test, *p* < 0.0001]. FM: fish meal; CBM: the chicken by-product meal (CBM)-substituted diets; MM: the meat meal (MM)-substituted diets; TBM: the tuna by-product meal (TBM)-substituted diets; 25%: dietary 25% FM substitutions; 50%: dietary 50% FM substitutions; Con: 55% FM-based diet; CBM25J: dietary 25% FM replacement with CBM with 22% jack mackerel meal (JMM) inclusion; CBM50J: dietary 50% FM replacement with CBM with 22% JMM inclusion; MM25J: dietary 25% FM replacement with MM with 22% JMM inclusion; MM50J: dietary 50% FM replacement with MM with 22% JMM inclusion; TBM25J: dietary 25% FM replacement with TBM with 22% JMM inclusion; TBM50J: dietary 50% FM replacement with TBM meal with 22% JMM inclusion. The different uppercase under numerical values indicate significant differences (*p* < 0.05) based on two-way ANOVA analysis. The different lowercase letters under numerical values indicate significant differences (*p* < 0.05) based on Duncan’s multiple comparisons.

**Figure 2:**
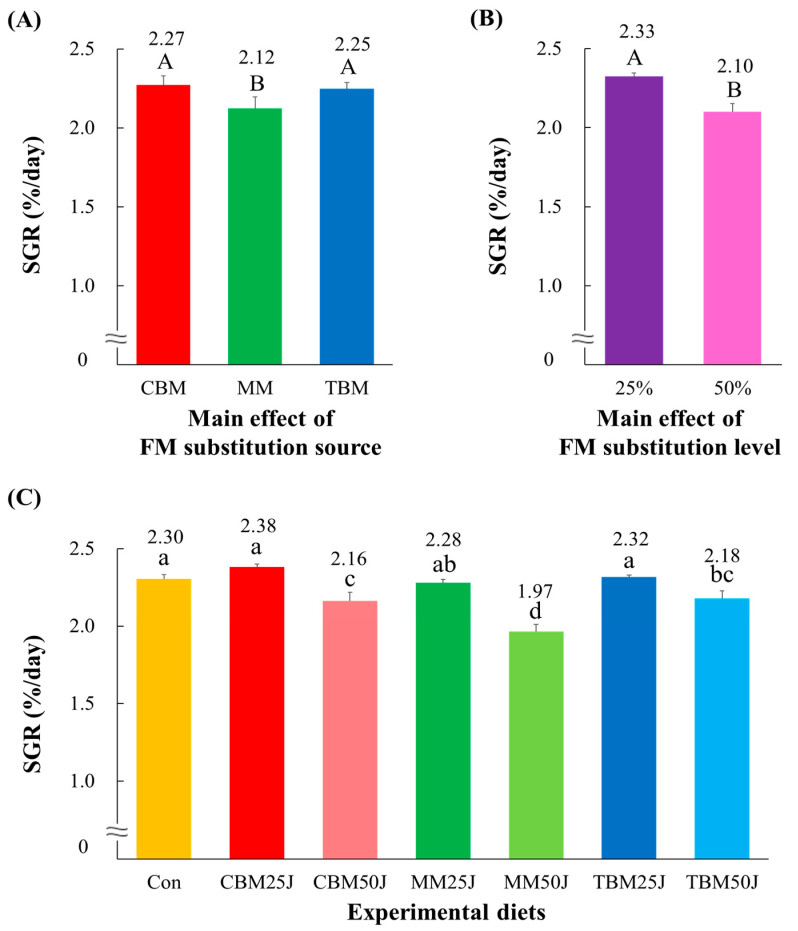
Specific growth rate (SGR, %/day) of rockfish (*Sebastes schlegeli*) fed the experimental diets for 8 weeks (mean of triplicate ± SE) [(**A**); the main effect of FM substitution source, *p* < 0.004, (**B**); the main effect of FM substitution level, *p* < 0.0001, and (**C**); Duncan’s multiple range test, *p* < 0.0001]. FM: fish meal; CBM: the chicken by-product meal (CBM)-substituted diets; MM: the meat meal (MM)-substituted diets; TBM: the tuna by-product meal (TBM)-substituted diets; 25%: dietary 25% FM substitutions; 50%: dietary 50% FM substitutions; Con: 55% FM-based diet; CBM25J: dietary 25% FM replacement with CBM with 22% jack mackerel meal (JMM) inclusion; CBM50J: dietary 50% FM replacement with CBM with 22% JMM inclusion; MM25J: dietary 25% FM replacement with MM with 22% JMM inclusion; MM50J: dietary 50% FM replacement with MM with 22% JMM inclusion; TBM25J: dietary 25% FM replacement with TBM with 22% JMM inclusion; TBM50J: dietary 50% FM replacement with TBM meal with 22% JMM inclusion. The different uppercase letters under numerical values indicate significant differences (*p* < 0.05) based on two-way ANOVA analysis. The different lowercase letters under numerical values indicate significant differences (*p* < 0.05) based on Duncan’s multiple comparisons.

The CBM- (21.5 g/fish and 2.27%/day, respectively) and TBM-substituted diets (21.0 g/fish and 2.25%/day) achieved significantly (*p* < 0.005 and *p* < 0.004, respectively) greater weight gain and SGR of rockfish than the MM-substituted diets (19.1 g/fish and 2.12%/day) (Figure 1A and Figure 2A, respectively). Moreover, dietary 25% FM substitutions (22.3 g/fish and 2.33%/day, respectively) achieved significantly (*p* < 0.0001 for both) greater weight gain and SGR of rockfish than dietary 50% FM substitutions (18.8 g/fish and 2.10%/day) (Figure 1B and Figure 2B, respectively). Weight gain and SGR of rockfish fed the Con, CBM25J, and TBM25J diets were significantly (*p* < 0.0001 for both) greater than those of rockfish fed the CBM50J, MM50J, and TBM50J diets but not significantly (*p* > 0.05) different from those of rockfish fed the MM25J diet (Figure 1C and Figure 2C, respectively). Rockfish fed the CBM25J diet achieved the greatest weight gain and SGR.

### 3.3. Feed Availability and Biological Indices of Rockfish

**Figure 3:**
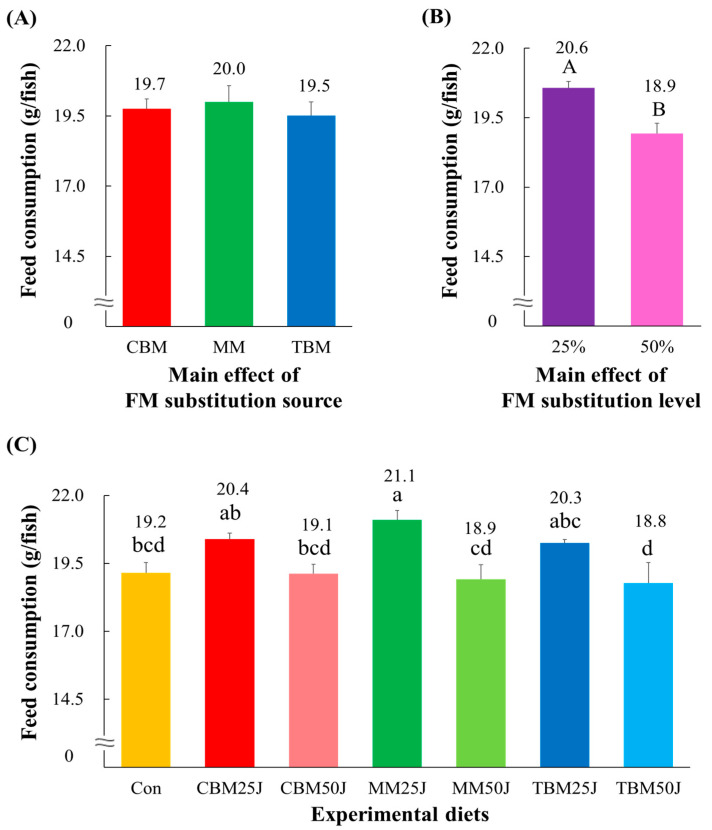
Feed consumption (g/fish) of rockfish (*Sebastes schlegeli*) fed the experimental diets for 8 weeks (mean of triplicate ± SE) [(**A**); the main effect of FM substitution source, *p* > 0.5, (**B**); the main effect of FM substitution level, *p* < 0.001, and (**C**); Duncan’s multiple range test, *p* < 0.01]. FM: fish meal; CBM: the chicken by-product meal (CBM)-substituted diets; MM: the meat meal (MM)-substituted diets; TBM: the tuna by-product meal (TBM)-substituted diets; 25%: dietary 25% FM substitutions; 50%: dietary 50% FM substitutions; Con: 55% FM-based diet; CBM25J: dietary 25% FM replacement with CBM with 22% jack mackerel meal (JMM) inclusion; CBM50J: dietary 50% FM replacement with CBM with 22% JMM inclusion; MM25J: dietary 25% FM replacement with MM with 22% JMM inclusion; MM50J: dietary 50% FM replacement with MM with 22% JMM inclusion; TBM25J: dietary 25% FM replacement with TBM with 22% JMM inclusion; TBM50J: dietary 50% FM replacement with TBM meal with 22% JMM inclusion. The different uppercase letters under numerical values indicate significant differences (*p* < 0.05) based on two-way ANOVA analysis. The different lowercase letters under numerical values indicate significant differences (*p* < 0.05) based on Duncan’s multiple comparisons.

None of the feed consumption of rockfish was significantly (*p* > 0.5) altered by the dietary substitution source (Figure 3A). Dietary 25% FM substitutions (20.6 g/fish) exhibited significantly (*p* < 0.001) higher feed consumption by rockfish than dietary 50% FM substitutions (18.9 g/fish) (Figure 3B). The feed consumption of rockfish fed the MM25J diet was significantly (*p* < 0.01) higher than that of rockfish fed the Con, CBM50J, MM50J, and TBM50J diets but not significantly (*p* > 0.05) different from that of rockfish fed CBM25J and TBM25J diets (Figure 3C).

The CBM- (1.09, 2.10, and 37.08%, respectively) and TBM-substituted diets (1.09, 2.10, and 36.45%) achieved significantly (*p* < 0.004, *p* < 0.003, and *p* < 0.01, respectively) higher FE, PER, and PR of rockfish than the MM-substituted diets (0.96, 1.86, and 32.52%) (Table 4). Moreover, dietary 25% FM substitutions (1.09 and 2.10) achieved significantly (*p* < 0.02 and *p* < 0.008, respectively) higher FE and PER of rockfish than dietary 50% FM substitutions (1.00 and 1.93). FE and PER of rockfish fed the Con and CBM25J diets were significantly (*p* < 0.001 for both) higher than those of rockfish fed the CBM50J, MM25J, and MM50J diets but not significantly (*p* > 0.05) different from those of rockfish fed the TBM25J and TBM50J diets. The PR of rockfish fed the Con diet was significantly (*p* < 0.005) higher than that of rockfish fed the CBM50J, MM25J, MM50J, TBM25J, and TBM50J diets but not significantly (*p* > 0.05) different from that of rockfish fed the CBM25J diet.

The biological indices (CF, VSI, and HSI) of rockfish were not significantly (*p* > 0.05) altered by either the dietary FM substitution source or substitution level.

### 3.4. Biochemical Composition of the Whole Body of Rockfish

No part of the proximate composition (moisture, crude protein, crude lipid, and ash content) of the whole body of rockfish was significantly (*p* > 0.05) altered by either the dietary substitution source or substitution level (Table 5).

None of the AA profiles of the whole body of rockfish was significantly (*p* > 0.05) affected by either dietary substitution source or substitution level (Table 6).

**Figure 4:**
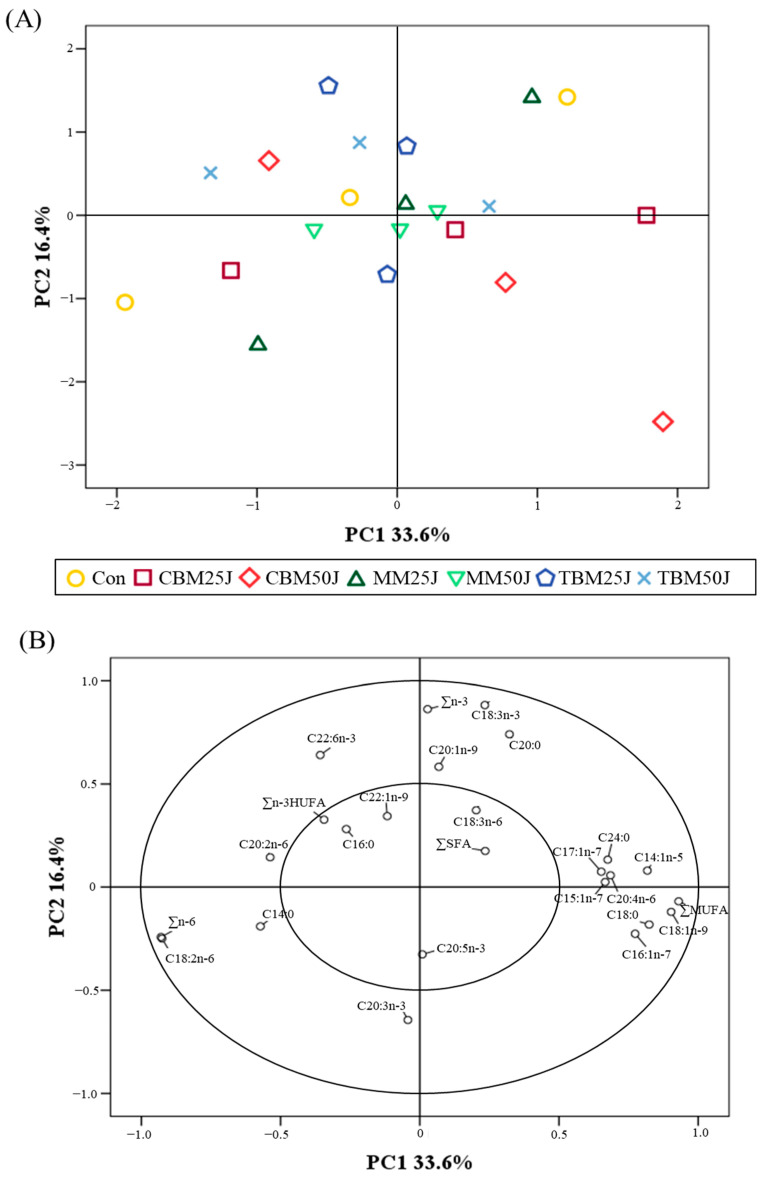
Principle component analysis (PCA) score plot (**A**) and correlation loadings plot (**B**) for the whole-body rockfish fed the experimental diet.

PCA score plot illustrated no clustering of FA profiles of the whole-body rockfish (Figure 4A). The 50% variation in the PCA model was explained by the first principal component (PC1) at 33.6% and the second principal component (PC2) at 16.4% (Figure 4B). The most significant variables in PC1 were ∑MUFA, oleic acid (C18:1n-9), myristoleic acid (C14:1n-5), stearic acid (C18:0), palmitoleic acid (C16:1n-7), arachidonic acid (C20:4n-6), lignoceric acid (C24:0), pentadecanoic acid (C15:1n-7), and heptadecenoic acid (C17:1n-7), while alpha-linolenic acid (C18:3n-3), ∑n-3, arachidic acid (C20:0), DHA, and eicosenoic acid (C20:1n-9) were identified as the most significant variables in PC2. Other variables had a minimal effect on the total variance in the PCA model.

### 3.5. Plasma and Serum Parameters of Rockfish

The MM- (169.0 U/L) and TBM-substituted diets (162.0 U/L) achieved significantly (*p* < 0.03) higher plasma AST of rockfish than the CBM-substituted diets (136.0 U/L) (Table 7). Moreover, the dietary 25% FM substitutions (296.9 mg/dL) achieved significantly (*p* < 0.05) higher T-CHO of rockfish than the dietary 50% FM substitutions (262.4 mg/dL). Nevertheless, the plasma parameters of rockfish were not significantly (*p* > 0.05) different among dietary treatments. None of the serum lysozyme activity and SOD of rockfish was significantly (*p* > 0.05) altered by either the dietary FM substitution source or substitution level.

### 3.6. Results of Economic Analysis of the Study

The price of the TBM25J diet (USD 1.64/kg) was the highest among the experimental diets, followed by the Con (USD 1.63/kg), MM25J (USD 1.60/kg), CBM25J (USD 1.59/kg), TBM50J (USD 1.52/kg), MM50J (USD 1.44/kg), and CBM50J (USD 1.42/kg) diets (Table 8). The MM-substituted diet (USD 1.60/kg) achieved significantly (*p* < 0.004) higher ECR than the CBM- (USD 1.39/kg) and TBM-substituted diets (USD 1.46/kg). However, the dietary substitution level did not significantly (*p* > 0.7) affect the ECR. The ECR of the MM50J diet was significantly (*p* < 0.02) higher than that of the Con, CBM25J, CBM50J, and TBM50J diets. The CBM- (USD 0.96/fish) and TBM-substituted diets (USD 0.95/fish) attained significantly (*p* < 0.006) higher EPI than the MM-substituted diet (USD 0.88/fish). Furthermore, dietary 25% FM substitutions (USD 0.99/fish) attained significantly (*p* < 0.0001) higher EPI than dietary 50% FM substitutions (USD 0.88/fish). The EPI of the CBM25J diet was significantly (*p* < 0.0001) higher than that of the CBM50J, MM50J, and TBM50J diets, but not significantly (*p* > 0.05) different from that of the Con, MM25J, and TBM25J diets.

## 4. Discussion

The substitutability of a replacer for FM in fish diets can vary profoundly depending on the quality of the replacer and the FM replacement level [40,41]. The CBM- and TBM-substituted diets resulted in superior weight gain and SGR of rockfish compared to the MM-substituted diets in the current experiment, suggesting that CBM and TBM could be more efficient as the FM replacers than MM in rockfish diets. Similarly, superior and comparable weight gain and SGR were obtained in olive flounder fed the diets replacing 30% FM with TBM and CBM and hydrolyzed chicken offal meal, respectively, compared to fish fed a 65% FM-based diet, but the growth performance of fish fed a diet replacing 30% FM with MM was inferior to fish fed a 65% FM-based diet [42]. Likewise, 50% FM could be substituted with CBM and TBM, whereas MM could substitute FM up to 25% without a reduction in weight gain and SGR of olive flounder when fish were provided with a 60% FM-basal diet or one of the diets substituting 25% and 50% FM with CBM, TBM, and MM with JMM inclusion [43]. The poorer growth performance (weight gain and SGR) of rockfish fed the MM-substituted diets compared to rockfish fed the CBM- and TBM-substituted diets might be attributed to the higher carbohydrate content in the former in this study. The MM25J and MM50J diets contained 24.6% and 26.7% carbohydrate content, respectively, which were higher than all other experimental diets (Con diet: 23.6% carbohydrates, CBM25J diet: 22.6%, CBM50J diet: 22.1%, TBM25J diet: 22.2%, and TBM50J diet: 21.6%) (Table 1). In general, carnivorous fish, including rockfish, have a poor utilization of dietary carbohydrates because of their low glucose metabolism [44,45]. Likewise, increased corn starch levels in diets led to linearly decreased growth performance of largemouth bass (*Micropterus salmoides*) when largemouth bass were supplied with diets containing 5%, 10%, 15%, 20%, and 25% corn starch [46]. Similarly, grouper (*Epinephelus akaara*) fed a diet containing 6% corn starch exhibited the best weight gain, but dietary elevated corn starch lowered weight gain when grouper were provided with diets containing 0%, 6%, 12%, 18%, 24%, and 30% corn starch [47].

The comparable weight gain and SGR of rockfish fed the CBM25J, MM25J, and TBM25J diets compared to rockfish fed the Con diet in this experiment also implied that 25% FM could be substituted with all alternative animal proteins in the rockfish diets with JMM inclusion without triggering any adverse effect on growth performance. Likewise, FM substitution with diverse proteins in diets supplemented with feed enhancers and/or stimulants achieved comparable growth performance to fish fed the FM-basal diets [48,49,50].

Dietary 25% FM substitutions attained greater weight gain, SGR, and feed consumption of rockfish compared to dietary 50% FM substitutions in this experiment. Similarly, increased FM replacement levels with an alternative protein source in fish diets commonly led to reduced feed intake and eventually deteriorated the growth performance of black sea turbot (*Psetta maeotica*) [51] and orange-spotted grouper (*Epinephelus coioides*) [52]. In particular, elevated FM substitution levels in the MM- and TBM-substituted diets led to a remarkable reduction in feed consumption by rockfish in this study. However, the feed consumption of rockfish fed the CBM25J diet was slightly, but not significantly, higher than that of fish fed CBM50J and Con diets. This could explain why the dietary 50% FM substitutions led to inferior weight gain and SGR compared to the dietary 25% FM substitutions in this experiment. Likewise, the reduced feed consumption of gilthead seabream (*Sparus aurata*) [53] and olive flounder [21] led to decreased growth performance when dietary FM replacement levels with poultry by-product meal (PBM) and tuna muscle by-product powder, respectively, were increased.

The greatest weight gain and SGR observed in rockfish fed the CBM25J diet in the present experiment might indicate that CBM was the most appropriate replacer for FM in rockfish feed, especially when 25% and 50% FM were replaced by various animal proteins (CBM, MM, and TBM) in diets with JMM inclusion. This could be demonstrated by the fact that CBM, PBM, and spray-dried poultry plasma containing high amounts of carnosine and anserine (potent antioxidants) could enhance the growth performance and FE of animals, such as fish and companion animals [54]. CBM, exclusively produced from chicken processing plants, is one type of PBM that is inexpensive and contains an acceptable crude protein level with limiting AA [55,56] and ∑n-3 HUFA [51,57]. However, Nandakumar et al. [10] reported that the substitutability of chicken waste meal for FM protein was limited to 10% in Asian seabass feed due to reduced feed consumption when fish were supplied with a 35% FM-basal diet or one of the diets replacing 5%, 10%, 15%, and 20% FM protein with chicken waste meal.

Although rockfish fed the CBM25J diet achieved the greatest growth performance (weight gain and SGR), JMM (USD 2.61/kg) is one of the most expensive FM sources. Therefore, its economic value and feasibility should be considered in practical feeding. Diet prices tended to decrease with increased FM substitution with alternative protein sources in this study. Furthermore, the CBM25J diet led to the highest EPI, suggesting that the CBM25J diet is expected to bring about the greatest economic return to rockfish farmers.

In the FAO/WHO ideal protein model, high-quality protein can be defined as having ∑EAAs/∑TAAs of approximately 40% and ∑EAAs/∑NEAAs of approximately 60% or more [58]. In this study, the ∑EAAs/∑TAAs ranged from 43% in the MM50J diet to 48% in the TBM25J and TBM50J diets, and the ∑EAAs/∑NEAAs ranged from 74% in the MM50J diet to 92% in the TBM25J and TBM50J diets (Table 2), indicating that the protein quality of all experimental diets did not cause any negative impact on growth performance of rockfish. Increased substitution of CBM, MM, and TBM for FM in diets with the inclusion of JMM led to increased arginine, leucine, and tryptophan in the CBM-substituted diets; arginine in the MM-substituted diets; and arginine, histidine, isoleucine, phenylalanine, and tryptophan in the TBM-substituted diets. However, when compared with the same level of FM replacement, all EAAs, except for arginine in the CBM- and TBM-substituted diets, were higher than those in the MM-substituted diets. This was probably one of the reasons for the inferior growth of rockfish fed the MM-substituted diets compared to fish fed the CBM- and TBM-substituted diets in this study. In particular, lysine and methionine are the limiting AA, and deficiencies in these AA in fish feed can lead to reduced growth, FE, protein synthesis, energy metabolism, and nitrogen retention [59,60,61]. The lysine requirement (2.99% of the diet) of rockfish [38] was fulfilled in all experimental diets, but the methionine requirement (1.37% of the diet in the presence of 0.12% cysteine) [39] was not met in most of the experimental diets (the CBM25J, CBM50J, MM25J, MM50J, and TBM50J diets), except for the Con and TBM25J diets. Previous studies have revealed that cysteine could spare approximately 40–50% of the dietary methionine requirement for orange-spotted grouper and yellowtail kingfish (*Seriola lalandi*) [59,62]. The cysteine content ranged from 0.60% in the MM50J diet to 0.82% in the Con diet in the present experiment, notably higher than the 0.12% cysteine of the diet reported in Yan et al. [39]’s study. Hence, the relatively low methionine in the experimental diets did not negatively impact the growth of rockfish.

Increased FM replacement levels in diets led to decreased ∑n-3 HUFA. This could be another reason why the CBM50J and MM50J diets resulted in inferior growth performance of rockfish compared to the Con diet. Likewise, weight gain and SGR of black seabream (*Acanthopagrus schlegeli*) fed a diet containing 1.75% ∑n-3 HUFA were the highest, but a dietary decrease in ∑n-3 HUFA (0.23%, 0.87%, and 1.29%) lowered growth performance when juvenile black seabreams were supplied with one of the diets containing 0.23%, 0.87%, 1.29%, 1.75%, and 2.53% ∑n-3 HUFA [63]. Similarly, the SGR of large yellow croaker (*Larimichthys crocea*) fed a diet containing 0.98% ∑n-3 HUFA was the highest, but dietary decreased ∑n-3 HUFA (0.15% and 0.60%) lowered the SGR when juvenile fish were supplied with one of the diets containing 0.15%, 0.60%, 0.98%, 1.37%, 1.79%, and 2.25% ∑n-3 HUFA [64].

Increased FM substitution levels in the CBM- and MM-substituted diets deteriorated the FE and PER of rockfish. Similarly, elevated FM substitution levels in low FM diets lowered the feed utilization of gibel carp (*Carassius auratus gibelio*) [65], olive flounder [66], and red drum (*Sciaenops ocellatus*) [67]. However, no remarkable difference in feed utilization (FE, PER, and PR) of rockfish fed the TBM25J and TBM50J diets in this experiment might indicate that the poorer weight gain and SGR of rockfish fed the TBM50J diet compared to rockfish fed the TBM25J diet resulted directly from the lower feed consumption of fish fed the former.

The CF of fish comes from the response of fish to external influences, such as the quality and quantity of nutrients, pathogens, and diseases [68]. The VSI and HSI are among the indicators used to determine dietary nutrient utilization and fish health [69]. None of the biological indices of rockfish was different among dietary treatments, suggesting that 25% and 50% FM substitution with CBM, MM, and TBM in diets with JMM inclusion did not adversely impact the biological indices of rockfish in the current experiment. Similarly, dietary FM replacement with diverse animal by-products (TBM, CBM, hydrolyzed chicken offal meal, MM, meat and bone meal, and blood meal), meat and bone meal, and PBM did not affect the biological indices of olive flounder [42], gibel carp [65], and humpback grouper (*Cromileptes altivelis*) [57], respectively.

The proximate composition and AA profiles of the whole-body rockfish were not different among dietary treatments in the current study. Furthermore, the FA profiles of the whole-body rockfish showed no clustering or other trends in the PCA model, indicating that FA profiles of the whole-body rockfish were not affected by dietary treatments. These findings indicated that 25% and 50% FM replacement by CBM, MM, and TBM in diets with JMM inclusion did not adversely affect the biochemical composition of the whole-body rockfish. Likewise, the proximate composition of red sea bream was not altered by different levels of FM substitution with porcine blood meal [70]. Kim et al. [17] also demonstrated that increased FM substitution levels with TBM in diets did not change the moisture, crude protein, and AA profiles of rockfish. Similarly, the chemical composition and FA profiles of silver perch (*Bidyanus bidyanus*) were not impacted by various levels of FM substitution with MM [71]. In addition, the biochemical composition of GIFT tilapia and red sea bream were not altered by the dietary inclusion of feed stimulants [72,73]. In particular, there are no discernible differences in the AA profiles of the whole-body rockfish because proteins in the body are synthesized from the genetic information of DNA [74].

Plasma parameters, except for plasma AST and T-CHO of rockfish, were unaffected by the dietary FM substitution source and level in this experiment. Likewise, dietary FM substitution with PBM and TBM did not influence the plasma chemistry of Nile tilapia and spotted rose snapper [75,76], respectively. Lysozyme is a vital defense molecule of the innate immune system, which is essential in mediating protection against microbial invasion [77]. SOD is also known to have major enzymatic activity, causing oxidative stress due to an imbalance between the oxidizing process and the antioxidant defense systems of an organism, resulting in tissue damage [78,79]. Serum lysozyme activity and SOD of rockfish were not different among dietary treatments in the current experiment. No discernible differences in plasma and serum parameters of rockfish in this experiment suggested that dietary treatments did not induce any adverse impact on the plasma and serum parameters of rockfish. Similarly, dietary FM substitution with fermented TBM did not bring about any difference in serum parameters of olive flounder [20]. In addition, lysozyme activity and SOD of olive flounder were not changed by supplementation of tilapia hydrolysate and krill hydrolysate in diets replacing 50% FM with soy protein concentrate [49]. However, lysozyme activity and SOD of olive flounder were affected by dietary FM replacement with various animal proteins [42] or the inclusion of feed stimulants and enhancers in low FM diets [50]. Both studies assumed that these differences might be associated with the growth of olive flounder because fish with poor growth performance commonly led to reduced lysozyme activity and SOD.

These results proved the effectiveness of CBM, TBM, and MM as FM replacers with JMM inclusion in the rockfish diets, highlighting their potential use for broader application. Furthermore, the findings can contribute to developing more sustainable and cost-effective dietary options for rockfish, thereby enhancing productivity and advancing aquaculture practices for this species. However, commercial-scale feeding trials are needed to evaluate the feasibility of replacing FM with various animal proteins in the rockfish feed in the long term.

## 5. Conclusions

The CBM- and TBM-substituted diets attained greater weight gain and SGR compared to the MM-substituted diets. Furthermore, dietary 25% FM substitutions attained greater weight gain, SGR, feed consumption, FE, and PER of rockfish than dietary 50% FM substitutions. Moreover, 25% FM replacement with CBM, MM, and TBM with 22% JMM inclusion in the 55% FM-based diet could be made without inducing any adverse impacts on the growth performance, feed consumption, biochemical composition, and blood chemistry of rockfish. The greatest weight gain and SGR were obtained in rockfish fed the CBM25J diet. Furthermore, the CBM25J diet produced the highest EPI. Therefore, the CBM25J diet is the most highly recommended diet for rockfish farmers based on weight gain, SGR, and EPI.

## Figures and Tables

**Table 2:** Amino acid (% of the diet) profiles of the experimental diets for rockfish (*Sebastes schlegeli*).

	Main Ingredients						Experimental Diets						
	FM	JMM	CBM	MM	TBM	Requirement	Con	CBM25J	CBM50J	MM25J	MM50J	TBM25J	TBM50J
Essential amino acids (EAAs, %)													
Arginine	4.02	4.19	4.14	5.71	3.53		2.91	3.08	3.12	3.15	3.22	2.96	2.99
Histidine	1.61	3.03	1.29	1.61	1.88		1.21	1.34	1.31	1.33	1.24	1.45	1.48
Isoleucine	2.48	2.90	1.95	1.99	2.41		1.60	1.71	1.66	1.66	1.55	1.84	2.01
Leucine	5.23	5.29	4.08	4.42	4.35		3.71	3.46	3.48	3.28	3.15	3.70	3.65
Lysine	5.53	5.96	3.92	4.13	4.54	2.99 ^a^	3.81	3.25	3.13	3.17	3.10	3.87	3.72
Methionine	2.01	2.10	1.27	1.27	1.39	1.37 ^b^	1.61	1.24	1.19	1.20	1.11	1.43	1.30
Phenylalanine	2.77	2.86	2.22	2.50	2.38		2.06	1.95	1.84	1.87	1.81	2.11	2.20
Threonine	3.14	3.28	2.43	2.42	2.57		2.24	2.06	1.97	1.94	1.79	2.10	2.06
Tryptophan	0.45	1.22	0.47	0.38	0.46		0.31	0.42	0.46	0.36	0.32	0.41	0.44
Valine	3.04	3.54	2.58	2.99	2.99		2.17	2.33	2.28	2.26	2.15	2.47	2.53
∑EAAs ^c^	30.28	34.37	24.35	27.42	26.50		21.63	20.84	20.44	20.22	19.44	22.34	22.38
Non-essential amino acids (NEAAs, %)													
Alanine	4.53	4.57	4.31	6.46	3.97		3.10	3.12	3.25	3.24	3.38	3.13	3.19
Aspartic acid	6.40	6.69	4.87	5.57	5.21		4.76	4.36	4.25	4.09	3.89	4.56	4.42
Cysteine	0.86	0.90	0.75	0.61	0.65	0.12 ^b^	0.82	0.81	0.82	0.69	0.60	0.74	0.71
Glutamic acid	8.95	9.28	8.22	9.73	7.30		7.09	7.19	7.34	6.99	6.91	7.08	6.98
Glycine	3.83	4.39	6.05	11.94	4.25		2.65	3.51	3.84	4.15	4.83	3.06	3.17
Proline	2.82	3.05	3.94	7.38	2.84		2.26	2.58	2.77	2.89	3.49	2.30	2.37
Serine	2.94	3.05	2.46	2.90	2.36		2.25	2.15	2.10	2.04	2.01	2.13	2.10
Tyrosine	2.06	2.04	1.44	1.44	1.49		1.40	1.16	1.12	1.14	1.06	1.19	1.16
∑NEAAs ^d^	32.39	33.97	32.04	46.03	28.07		24.33	24.88	25.49	25.23	26.17	24.19	24.30
∑TAAs ^e^	62.67	68.34	56.39	73.45	54.57		45.96	45.72	45.93	45.45	45.61	46.53	46.68
∑EAAs/∑TAAs	0.48	0.50	0.43	0.37	0.49		0.47	0.46	0.45	0.44	0.43	0.48	0.48
∑EAAs/∑NEAAs	0.93	1.01	0.76	0.60	0.94		0.89	0.84	0.80	0.80	0.74	0.92	0.92

FM: fish meal; JMM: jack mackerel meal; CBM: chicken by-product meal; MM: meat meal; TBM: tuna by-product meal; Con: 55% FM-based diet; CBM25J: dietary 25% FM replacement with CBM with 22% JMM inclusion; CBM50J: dietary 50% FM replacement with CBM with 22% JMM inclusion; MM25J: dietary 25% FM replacement with MM with 22% JMM inclusion; MM50J: dietary 50% FM replacement with MM with 22% JMM inclusion; TBM25J: dietary 25% FM replacement with TBM with 22% JMM inclusion; TBM50J: dietary 50% FM replacement with TBM with 22% JMM inclusion. ^a,b^ Data were obtained from Yan et al. [38,39]’s studies, respectively. ^c^ ∑EAAs: total essential amino acids. ^d^ ∑NEAAs: total non-essential amino acids. ^e^ ∑TAAs: total amino acids.

**Table 3:** Fatty acid (% of total fatty acids) profiles of the experimental diets for rockfish (*Sebastes schlegeli*).

	Main Ingredients					Experimental Diets						
Fatty Acid (%)	FM	JMM	CBM	MM	TBM	Con	CBM25J	CBM50J	MM25J	MM50J	TBM25J	TBM50J
C14:0	6.41	5.01	0.97	2.43	4.22	1.69	1.39	1.36	1.50	1.31	1.65	1.62
C16:0	23.34	21.42	28.12	28.08	27.36	15.19	17.05	17.74	17.01	17.69	16.84	16.98
C18:0	5.01	7.44	8.71	14.99	8.06	4.23	4.97	5.02	5.93	6.68	4.83	5.09
C20:0	0.32	0.32	0.17	0.23	0.69	0.26	0.34	0.34	0.27	0.35	0.30	0.32
C24:0	2.56	3.31	0.04	0.03	1.26	0.66	0.61	0.47	0.59	0.39	0.65	0.57
∑SFA ^a^	37.64	37.50	38.01	45.76	41.59	22.03	24.36	24.93	25.30	26.42	24.27	24.58
C14:1n-5	0.13	0.14	0.24	0.05	0.05	0.01	0.04	0.07	0.04	0.05	0.02	0.04
C15:1n-7	0.07	0.07	0.27	0.06	0.13	0.02	0.02	0.02	0.02	0.02	0.03	0.04
C16:1n-7	6.73	6.74	5.78	3.05	5.65	2.26	2.24	2.18	2.18	2.04	2.22	2.19
C17:1n-7	0.81	0.86	0.19	0.45	0.75	0.12	0.16	0.12	0.17	0.16	0.16	0.14
C18:1n-9	15.23	20.36	48.02	45.46	23.47	22.38	28.21	30.51	27.79	29.06	25.85	26.38
C20:1n-9	4.17	1.80	1.05	1.36	2.30	2.11	0.88	0.85	1.03	0.94	1.21	1.05
C22:1n-9	0.45	0.83	0.06	0.03	2.16	0.28	0.24	0.22	0.25	0.25	0.29	0.31
∑MUFA ^b^	27.59	30.80	55.61	50.46	34.51	27.18	31.79	33.97	31.48	32.52	29.78	30.15
C18:2n-6	4.17	1.73	4.86	2.30	2.37	35.72	31.81	31.88	31.68	31.02	31.75	31.68
C18:3n-3	0.57	0.76	0.06	0.03	0.80	4.43	3.67	2.07	3.59	3.54	4.52	4.42
C18:3n-6	0.23	0.10	0.02	0.04	0.32	0.06	0.06	0.04	0.05	0.05	0.06	0.06
C20:2n-6	0.08	0.17	0.08	0.09	0.29	0.13	0.12	0.12	0.12	0.11	0.14	0.15
C20:3n-3	0.08					0.03	0.03	0.03	0.04	0.03	0.04	0.05
C20:4n-6	1.14	0.69	0.36	0.15	1.31	0.36	0.33	0.31	0.30	0.24	0.42	0.44
C20:5n-3	10.54	10.73	0.13	0.06	4.58	4.53	3.59	3.02	3.49	2.76	3.86	3.36
C22:6n-3	13.30	15.40	0.37	0.02	10.48	4.61	3.52	3.01	3.24	2.68	4.17	4.07
∑n-3 HUFA ^c^	23.92	26.13	0.50	0.08	15.06	9.17	7.14	6.06	6.77	5.47	8.07	7.48
∑n-3 ^d^	24.49	26.89	0.56	0.11	15.86	13.60	10.81	8.13	10.36	9.01	12.59	11.90
∑n-6 ^e^	5.62	2.69	5.32	2.58	4.29	36.27	32.32	32.35	32.15	31.42	32.37	32.33
∑n-3/∑n-6	4.36	10.00	0.11	0.04	3.70	0.37	0.33	0.25	0.32	0.29	0.39	0.37
Unknown	4.66	2.12	0.50	1.09	3.75	0.92	0.72	0.62	0.71	0.63	0.99	1.04

FM: fish meal; JMM: jack mackerel meal; CBM: chicken by-product meal; MM: meat meal; TBM: tuna by-product meal; Con: 55% FM-based diet; CBM25J: dietary 25% FM replacement with CBM with 22% JMM inclusion; CBM50J: dietary 50% FM replacement with CBM with 22% JMM inclusion; MM25J: dietary 25% FM replacement with MM with 22% JMM inclusion; MM50J: dietary 50% FM replacement with MM with 22% JMM inclusion; TBM25J: dietary 25% FM replacement with TBM with 22% JMM inclusion; TBM50J: dietary 50% FM replacement with TBM with 22% JMM inclusion. ^a^ ∑SFAs: total saturated fatty acids. ^b^ ∑MUFAs: total monounsaturated fatty acids. ^c^ ∑n-3 HUFAs: total n-3 highly unsaturated fatty acids. ^d^ ∑n-3: total n-3 fatty acids. ^e^ ∑n-6: total n-6 fatty acids.

**Table 4:** Survival (%), feed efficiency (FE), protein efficiency ratio (PER), protein retention (PR, %), condition factor (CF), viscerosomatic index (VSI, %), and hepatosomatic index (HSI, %) of rockfish (*Sebastes schlegeli*) fed the experimental diets for 8 weeks.

Experimental Diets	Initial Weight (g/Fish)	Final Weight (g/Fish)	Survival (%)	FE ^a^	PER ^b^	PR ^c^ (%)	CF ^d^	VSI ^e^ (%)	HIS ^f^ (%)
Con	8.3 ± 0.07	30.1 ± 0.25 ^ab^	100.0 ± 0.00	1.14 ± 0.006 ^a^	2.21 ± 0.012 ^a^	41.03 ± 1.811 ^a^	1.65 ± 0.032	9.86 ± 0.271	2.79 ± 0.118
CBM25J	8.3 ± 0.05	31.6 ± 0.39 ^a^	98.7 ± 1.33	1.15 ± 0.035 ^a^	2.21 ± 0.058 ^a^	38.41 ± 1.346 ^ab^	1.62 ± 0.030	10.20 ± 0.309	2.89 ± 0.021
CBM50J	8.3 ± 0.09	28.0 ± 0.96 ^c^	98.7 ± 1.33	1.03 ± 0.032 ^b^	1.98 ± 0.063 ^b^	35.75 ± 0.889 ^b^	1.60 ± 0.040	9.46 ± 0.179	2.57 ± 0.148
MM25J	8.3 ± 0.05	29.8 ± 0.56 ^ab^	96.0 ± 2.31	1.03 ± 0.043 ^b^	1.98 ± 0.080 ^b^	34.44 ± 1.494 ^bc^	1.63 ± 0.050	9.47 ± 0.112	2.84 ± 0.113
MM50J	8.3 ± 0.05	25.1 ± 0.48 ^d^	100.0 ± 0.00	0.89 ± 0.050 ^c^	1.73 ± 0.097 ^c^	30.60 ± 2.074 ^c^	1.54 ± 0.016	9.65 ± 0.161	3.10 ± 0.030
TBM25J	8.3 ± 0.07	30.4 ± 0.26 ^a^	100.0 ± 0.00	1.09 ± 0.010 ^ab^	2.12 ± 0.020 ^ab^	36.39 ± 1.155 ^b^	1.60 ± 0.009	9.77 ± 0.423	2.51 ± 0.251
TBM50J	8.4 ± 0.03	28.3 ± 0.73 ^bc^	97.3 ± 2.67	1.09 ± 0.025 ^ab^	2.08 ± 0.043 ^ab^	36.51 ± 0.241 ^b^	1.63 ± 0.018	9.98 ± 0.448	2.69 ± 0.191
*p*-value		*p* < 0.0001	*p* > 0.4	*p* < 0.001	*p* < 0.001	*p* < 0.005	*p* > 0.3	*p* > 0.5	*p* > 0.1
Main effect: substitution source									
CBM		29.8 ± 0.93 ^A^	98.7 ± 0.84	1.09 ± 0.034 ^A^	2.10 ± 0.063 ^A^	37.08 ± 0.935 ^A^	1.61 ± 0.023	9.83 ± 0.229	2.73 ± 0.097
MM		27.5 ± 1.10 ^B^	98.0 ± 1.37	0.96 ± 0.043 ^B^	1.86 ± 0.079 ^B^	32.52 ± 1.430 ^B^	1.59 ± 0.030	9.56 ± 0.097	2.97 ± 0.078
TBM		29.4 ± 0.57 ^A^	98.7 ± 1.33	1.09 ± 0.012 ^A^	2.10 ± 0.023 ^A^	36.45 ± 0.529 ^A^	1.61 ± 0.013	9.87 ± 0.279	2.60 ± 0.147
Main effect: substitution level									
25%		30.6 ± 0.41 ^A^	98.2 ± 1.19	1.09 ± 0.029 ^A^	2.10 ± 0.054 ^A^	36.41 ± 1.080	1.61 ± 0.022	9.81 ± 0.229	2.74 ± 0.122
50%		27.1 ± 0.78 ^B^	98.7 ± 1.16	1.00 ± 0.043 ^B^	1.93 ± 0.077 ^B^	34.28 ± 1.391	1.59 ± 0.023	9.70 ± 0.202	2.79 ± 0.129
Two-way ANOVA									
Substitution source		*p* < 0.005	*p* > 0.8	*p* < 0.004	*p* < 0.003	*p* < 0.01	*p* > 0.5	*p* > 0.7	*p* > 0.08
Substitution level		*p* < 0.0001	*p* > 0.7	*p* < 0.02	*p* < 0.008	*p* > 0.07	*p* > 0.3	*p* > 0.7	*p* > 0.6
Interaction		*p* > 0.1	*p* > 0.1	*p* > 0.1	*p* > 0.2	*p* > 0.3	*p* > 0.1	*p* > 0.4	*p* > 0.2

Con: 55% fish meal (FM)-based diet; CBM25J: dietary 25% FM replacement with chicken by-product meal (CBM) with 22% jack mackerel meal (JMM) inclusion; CBM50J: dietary 50% FM replacement with CBM with 22% JMM inclusion; MM25J: dietary 25% FM replacement with meat meal (MM) with 22% JMM inclusion; MM50J: dietary 50% FM replacement with MM with 22% JMM inclusion; TBM25J: dietary 25% FM replacement with tuna by-product meal (TBM) with 22% JMM inclusion; TBM50J: dietary 50% FM replacement with TBM with 22% JMM; CBM: the CBM-substituted diets; MM: the MM-substituted diets; TBM: the TBM-substituted diets; 25%: dietary 25% FM substitutions; 50%: dietary 50% FM substitutions. Values (means of triplicate ± SE) with different lowercase and uppercase letters indicated statistical differences (*p* < 0.05) based on Duncan’s multiple range test and two-way ANOVA analysis, respectively. ^a^ Feed efficiency (FE) = weight gain of fish (g)/total feed consumption (g). ^b^ Protein efficiency ratio (PER) = weight gain of fish (g)/protein consumption (g). ^c^ Protein retention (PR, %) = protein gain of fish (g) × 100/protein consumption (g). ^d^ Condition factor (CF) = body weight of fish (g) × 100/total length of fish (cm)^3^. ^e^ Viscerosomatic index (VSI, %) = viscera weight of fish (g) × 100/body weight of fish (g). ^f^ Hepatosomatic index (HSI, %) = liver weight of fish (g) × 100/body weight of fish (g).

**Table 5:** Proximate composition (%) of the whole body of rockfish (*Sebastes schlegeli*) fed the experimental diets replacing 25% and 50% fish meal with various animal by-product meals with jack mackerel meal inclusion for 8 weeks.

Experimental Diets	Moisture	Crude Protein	Crude Lipid	Ash
Con	69.9 ± 1.30	18.0 ± 0.62	8.6 ± 0.29	4.4 ± 0.12
CBM25J	69.7 ± 0.67	17.1 ± 0.12	7.9 ± 0.37	4.5 ± 0.24
CBM50J	70.8 ± 1.22	17.5 ± 0.37	6.4 ± 0.68	4.8 ± 0.15
MM25J	70.6 ± 1.63	17.1 ± 0.08	7.9 ± 0.68	4.6 ± 0.11
MM50J	70.6 ± 2.28	17.2 ± 0.25	7.0 ± 0.30	4.7 ± 0.27
TBM25J	70.4 ± 1.04	16.9 ± 0.38	7.4 ± 0.61	4.4 ± 0.44
TBM50J	68.3 ± 2.55	17.2 ± 0.23	7.9 ± 0.05	4.6 ± 0.47
*p*-value	*p* > 0.9	*p* > 0.4	*p* > 0.1	*p* > 0.9
Main effect: substitution source				
CBM	70.2 ± 0.67	17.3 ± 0.20	7.1 ± 0.48	4.6 ± 0.14
MM	70.6 ± 1.25	17.2 ± 0.12	7.5 ± 0.39	4.6 ± 0.13
TBM	69.4 ± 1.32	17.0 ± 0.21	7.7 ± 0.29	4.5 ± 0.29
Main effect: substitution level				
25%	70.3 ± 0.74	17.1 ± 0.15	7.7 ± 0.36	4.5 ± 0.18
50%	69.9 ± 1.37	17.3 ± 0.19	7.1 ± 0.38	4.7 ± 0.20
Two-way ANOVA				
Substitution source	*p* > 0.7	*p* > 0.6	*p* > 0.6	*p* > 0.8
Substitution level	*p* > 0.7	*p* > 0.2	*p* > 0.1	*p* > 0.4
Interaction	*p* > 0.6	*p* > 0.8	*p* > 0.1	*p* > 0.9

Con: 55% fish meal (FM)-based diet; CBM25J: dietary 25% FM replacement with chicken by-product meal (CBM) with 22% jack mackerel meal (JMM) inclusion; CBM50J: dietary 50% FM replacement with CBM with 22% JMM inclusion; MM25J: dietary 25% FM replacement with meat meal (MM) with 22% JMM inclusion; MM50J: dietary 50% FM replacement with MM with 22% JMM inclusion; TBM25J: dietary 25% FM replacement with tuna by-product meal (TBM) with 22% JMM inclusion; TBM50J: dietary 50% FM replacement with TBM with 22% JMM; CBM: the CBM-substituted diets; MM: the MM-substituted diets; TBM: the TBM-substituted diets; 25%: dietary 25% FM substitutions; 50%: dietary 50% FM substitutions. Values (means of triplicate ± SE) are presented.

**Table 6:** Amino acid (% of wet weight) profiles of the whole body of rockfish (*Sebastes schlegeli*) fed the experimental diets replacing 25% and 50% fish meal with various animal by-product meals with jack mackerel meal inclusion for 8 weeks.

	Experimental Diets								Main Effect: Substitution Source			Main Effect: Substitution Level		Two-Way ANOVA		
	Con	CBM25J	CBM50J	MM25J	MM50J	TBM25J	TBM50J	*p*-Value	CBM	MM	TBM	25%	50%	Substitution Source	Substitution Level	Interaction
Essential amino acids (%)																
Arginine	1.1 ± 0.03	1.1 ± 0.08	1.1 ± 0.02	1.1 ± 0.04	1.1 ± 0.06	1.1 ± 0.04	1.0 ± 0.01	*p* > 0.5	1.1 ± 0.04	1.1 ± 0.04	1.0 ± 0.03	1.1 ± 0.04	1.1 ± 0.03	*p* > 0.4	*p* > 0.4	*p* > 0.3
Histidine	0.4 ± 0.17	0.4 ± 0.06	0.4 ± 0.02	0.4 ± 0.07	0.4 ± 0.07	0.4 ± 0.06	0.4 ± 0.08	*p* > 0.9	0.4 ± 0.03	0.4 ± 0.04	0.4 ± 0.05	0.4 ± 0.04	0.4 ± 0.04	*p* > 0.9	*p* > 0.6	*p* > 0.9
Isoleucine	0.8 ± 0.12	0.7 ± 0.11	0.7 ± 0.08	0.7 ± 0.10	0.7 ± 0.10	0.7 ± 0.11	0.7 ± 0.07	*p* > 0.9	0.7 ± 0.06	0.7 ± 0.06	0.7 ± 0.06	0.7 ± 0.07	0.7 ± 0.05	*p* > 0.9	*p* > 0.5	*p* > 0.8
Leucine	1.3 ± 0.10	1.3 ± 0.10	1.2 ± 0.06	1.2 ± 0.06	1.2 ± 0.08	1.3 ± 0.10	1.1 ± 0.01	*p* > 0.8	1.2 ± 0.05	1.2 ± 0.04	1.2 ± 0.05	1.3 ± 0.05	1.2 ± 0.04	*p* > 0.8	*p* > 0.3	*p* > 0.5
Lysine	1.4 ± 0.10	1.5 ± 0.12	1.4 ± 0.08	1.4 ± 0.08	1.4 ± 0.11	1.5 ± 0.11	1.3 ± 0.01	*p* > 0.7	1.4 ± 0.07	1.4 ± 0.06	1.4 ± 0.06	1.4 ± 0.06	1.4 ± 0.06	*p* > 0.8	*p* > 0.3	*p* > 0.5
Methionine	0.6 ± 0.05	0.6 ± 0.05	0.6 ± 0.04	0.5 ± 0.02	0.6 ± 0.07	0.6 ± 0.05	0.6 ± 0.03	*p* > 0.7	0.6 ± 0.03	0.6 ± 0.04	0.6 ± 0.03	0.6 ± 0.03	0.6 ± 0.03	*p* > 0.9	*p* > 0.9	*p* > 0.2
Phenylalanine	0.7 ± 0.08	0.7 ± 0.04	0.7 ± 0.05	0.7 ± 0.03	0.7 ± 0.05	0.7 ± 0.05	0.7 ± 0.03	*p* > 0.9	0.7 ± 0.03	0.7 ± 0.03	0.7 ± 0.03	0.7 ± 0.03	0.7 ± 0.03	*p* > 0.9	*p* > 0.7	*p* > 0.7
Threonine	0.8 ± 0.04	0.8 ± 0.05	0.8 ± 0.04	0.8 ± 0.04	0.8 ± 0.06	0.8 ± 0.06	0.7 ± 0.02	*p* > 0.8	0.8 ± 0.03	0.8 ± 0.03	0.7 ± 0.03	0.8 ± 0.03	0.8 ± 0.03	*p* > 0.5	*p* > 0.5	*p* > 0.6
Tryptophan	0.1 ± 0.01	0.1 ± 0.02	0.1 ± 0.02	0.1 ± 0.01	0.1 ± 0.02	0.1 ± 0.02	0.1 ± 0.03	*p* > 0.9	0.1 ± 0.01	0.1 ± 0.01	0.1 ± 0.02	0.1 ± 0.01	0.1 ± 0.01	*p* > 0.8	*p* > 0.4	*p* > 0.8
Valine	0.9 ± 0.10	0.8 ± 0.01	0.8 ± 0.05	0.8 ± 0.04	0.8 ± 0.07	0.9 ± 0.10	0.7 ± 0.06	*p* > 0.8	0.8 ± 0.03	0.8 ± 0.04	0.8 ± 0.06	0.8 ± 0.04	0.8 ± 0.04	*p* > 0.9	*p* > 0.3	*p* > 0.6
Non-essential amino acids (%)																
Alanine	1.2 ± 0.06	1.2 ± 0.07	1.2 ± 0.04	1.2 ± 0.03	1.3 ± 0.08	1.2 ± 0.05	1.1 ± 0.01	*p* > 0.6	1.2 ± 0.04	1.2 ± 0.04	1.2 ± 0.03	1.2 ± 0.04	1.2 ± 0.04	*p* > 0.6	*p* > 0.7	*p* > 0.2
Aspartic acid	1.7 ± 0.12	1.7 ± 0.13	1.6 ± 0.08	1.7 ± 0.08	1.7 ± 0.11	1.7 ± 0.12	1.5 ± 0.02	*p* > 0.7	1.7 ± 0.07	1.7 ± 0.06	1.6 ± 0.07	1.7 ± 0.04	1.6 ± 0.06	*p* > 0.7	*p* > 0.4	*p* > 0.4
Cysteine	0.3 ± 0.03	0.2 ± 0.02	0.3 ± 0.03	0.2 ± 0.02	0.3 ± 0.03	0.2 ± 0.02	0.2 ± 0.01	*p* > 0.9	0.2 ± 0.02	0.2 ± 0.02	0.2 ± 0.01	0.2 ± 0.01	0.2 ± 0.02	*p* > 0.7	*p* > 0.9	*p* > 0.5
Glutamic acid	2.4 ± 0.12	2.4 ± 0.14	2.3 ± 0.12	2.4 ± 0.13	2.4 ± 0.17	2.4 ± 0.15	2.2 ± 0.22	*p* > 0.8	2.4 ± 0.09	2.4 ± 0.10	2.3 ± 0.13	2.4 ± 0.09	2.3 ± 0.11	*p* > 0.8	*p* > 0.4	*p* > 0.6
Glycine	1.5 ± 0.05	1.6 ± 0.12	1.5 ± 0.04	1.4 ± 0.08	1.6 ± 0.12	1.5 ± 0.04	1.5 ± 0.06	*p* > 0.6	1.5 ± 0.06	1.5 ± 0.07	1.5 ± 0.03	1.5 ± 0.06	1.5 ± 0.05	*p* > 0.7	*p* > 0.7	*p* > 0.2
Proline	0.8 ± 0.06	0.9 ± 0.10	0.8 ± 0.06	0.8 ± 0.05	0.9 ± 0.10	0.8 ± 0.04	0.8 ± 0.04	*p* > 0.9	0.8 ± 0.06	0.8 ± 0.05	0.8 ± 0.03	0.8 ± 0.05	0.8 ± 0.05	*p* > 0.7	*p* > 0.9	*p* > 0.6
Serine	0.7 ± 0.06	0.8 ± 0.07	0.8 ± 0.04	0.8 ± 0.05	0.8 ± 0.05	0.7 ± 0.04	0.7 ± 0.05	*p* > 0.3	0.8 ± 0.04	0.8 ± 0.04	0.7 ± 0.03	0.8 ± 0.04	0.8 ± 0.04	*p* > 0.1	*p* > 0.8	*p* > 0.4
Tyrosine	0.3 ± 0.03	0.4 ± 0.09	0.4 ± 0.05	0.4 ± 0.10	0.4 ± 0.09	0.4 ± 0.03	0.3 ± 0.04	*p* > 0.7	0.4 ± 0.04	0.4 ± 0.06	0.3 ± 0.02	0.4 ± 0.05	0.4 ± 0.04	*p* > 0.5	*p* > 0.7	*p* > 0.9

Con: 55% fish meal (FM)-based diet; CBM25J: dietary 25% FM replacement with chicken by-product meal (CBM) with 22% jack mackerel meal (JMM) inclusion; CBM50J: dietary 50% FM replacement with CBM with 22% JMM inclusion; MM25J: dietary 25% FM replacement with meat meal (MM) with 22% JMM inclusion; MM50J: dietary 50% FM replacement with MM with 22% JMM inclusion; TBM25J: dietary 25% FM replacement with tuna by-product meal (TBM) with 22% JMM inclusion; TBM50J: dietary 50% FM replacement with TBM with 22% JMM; CBM: the CBM-substituted diets; MM: the MM-substituted diets; TBM: the TBM-substituted diets; 25%: dietary 25% FM substitutions; 50%: dietary 50% FM substitutions. Values (means of triplicate ± SE) are presented.

**Table 7:** Plasma and serum parameters of rockfish (*Sebastes schlegeli*) fed the experimental diets replacing 25% and 50% fish meal with various animal by-product meals with jack mackemel meal inclusion for 8 weeks.

Experimental Diets	Plasma Parameters								Serum Parameters	
	AST (U/L)	ALT (U/L)	ALP (U/L)	T-BIL (mg/dL)	T-CHO (mg/dL)	TG (mg/dL)	TP (g/dL)	ALB (g/dL)	Lysozyme Activity (U/mL)	SOD (ng/mL)
Con	154.0 ± 8.89	29.7 ± 1.67	293.0 ± 14.29	1.1 ± 0.30	299.0 ± 21.08	358.7 ± 23.41	5.4 ± 0.20	1.5 ± 0.10	502.0 ± 24.04	3.6 ± 0.18
CBM25J	133.3 ± 12.45	33.0 ± 12.77	229.0 ± 50.90	0.7 ± 0.06	285.7 ± 24.70	407.0 ± 55.19	4.8 ± 0.21	1.3 ± 0.19	386.7 ± 43.81	3.6 ± 0.24
CBM50J	138.7 ± 10.33	23.3 ± 2.60	230.0 ± 39.72	0.9 ± 0.17	254.7 ± 23.82	393.3 ± 18.50	4.8 ± 0.17	1.4 ± 0.21	408.9 ± 51.14	3.8 ± 0.41
MM25J	154.7 ± 16.75	28.3 ± 4.33	361.3 ± 40.96	2.0 ± 0.78	323.3 ± 20.34	351.0 ± 30.53	5.9 ± 0.47	2.0 ± 0.21	398.3 ± 31.93	4.0 ± 0.25
MM50J	183.3 ± 5.36	34.3 ± 5.78	246.3 ± 36.09	1.5 ± 0.18	276.3 ± 9.96	436.7 ± 21.88	5.1 ± 0.26	1.5 ± 0.09	445.0 ± 25.66	4.0 ± 0.06
TBM25J	152.3 ± 4.98	34.0 ± 2.31	260.0 ± 27.06	0.9 ± 0.37	281.7 ± 17.90	466.3 ± 33.67	5.0 ± 0.27	1.3 ± 0.15	411.7 ± 79.91	3.7 ± 0.32
TBM50J	171.7 ± 9.39	29.0 ± 8.08	228.7 ± 27.72	1.8 ± 0.88	256.3 ± 12.88	472.3 ± 27.67	5.0 ± 0.77	1.5 ± 0.38	415.0 ± 31.22	3.8 ± 0.25
*p*-value	*p* > 0.05	*p* > 0.8	*p* > 0.1	*p* > 0.4	*p* > 0.2	*p* > 0.09	*p* > 0.5	*p* > 0.3	*p* > 0.7	*p* > 0.9
Main effect: substitution source										
CBM	136.0 ± 7.33 ^B^	28.2 ± 6.22	229.5 ± 28.87	0.8 ± 0.09	270.2 ± 16.84	400.2 ± 26.21	4.8 ± 0.12	1.4 ± 0.13	421.7 ± 21.08	3.7 ± 0.31
MM	169.0 ± 10.15 ^A^	31.3 ± 3.50	303.8 ± 35.46	1.8 ± 0.37	299.8 ± 14.60	393.7 ± 25.48	5.5 ± 0.29	1.7 ± 0.16	413.3 ± 38.38	3.8 ± 0.26
TBM	162.0 ± 6.42 ^A^	31.5 ± 3.92	244.3 ± 18.69	1.3 ± 0.47	269.0 ± 11.37	469.32 ± 19.53	5.0 ± 0.37	1.4 ± 0.19	437.3 ± 37.63	3.8 ± 0.20
Main effect: substitution level										
25%	146.8 ± 8.64	31.8 ± 4.95	283.4 ± 34.98	1.2 ± 0.40	296.9 ± 15.30 ^A^	408.1 ± 32.48	5.2 ± 0.29	1.6 ± 0.18	413.3 ± 38.39	3.6 ± 0.21
50%	164.6 ± 9.75	28.9 ± 4.12	235.0 ± 21.64	1.4 ± 0.36	262.4 ± 11.05 ^B^	434.1 ± 19.85	5.0 ± 0.30	1.5 ± 0.16	434.9 ± 24.55	3.9 ± 0.28
Two-way ANOVA										
Substitution source	*p* < 0.03	*p* > 0.8	*p* > 0.1	*p* > 0.1	*p* > 0.2	*p* > 0.08	*p* > 0.2	*p >* 0.2	*p* > 0.8	*p* > 0.9
Substitution level	*p* > 0.06	*p* > 0.6	*p* > 0.1	*p* > 0.6	*p* < 0.05	*p* > 0.3	*p* > 0.7	*p* > 0.6	*p* > 0.6	*p* > 0.4
Interaction	*p* > 0.5	*p* > 0.5	*p* > 0.3	*p* > 0.4	*p* > 0.8	*p* > 0.3	*p* > 0.6	*p* > 0.2	*p* > 0.9	*p* > 0.9

Con: 55% fish meal (FM)-based diet; CBM25J: dietary 25% FM replacement with chicken by-product meal (CBM) with 22% jack mackerel meal (JMM) inclusion; CBM50J: dietary 50% FM replacement with CBM with 22% JMM inclusion; MM25J: dietary 25% FM replacement with meat meal (MM) with 22% JMM inclusion; MM50J: dietary 50% FM replacement with MM with 22% JMM inclusion; TBM25J: dietary 25% FM replacement with tuna by-product meal (TBM) with 22% JMM inclusion; TBM50J: dietary 50% FM replacement with TBM with 22% JMM; CBM: the CBM-substituted diets; MM: the MM-substituted diets; TBM: the TBM-substituted diets; 25%: dietary 25% FM substitutions; 50%: dietary 50% FM substitutions. Values (means of triplicate ± SE) with different uppercase letters indicate statistical differences (*p* < 0.05) based on two-way ANOVA analysis.

**Table 8:** Economic parameters [diet price, economic conversion ratio (ECR), and economic profit index (EPI)] of the feeding trial.

Experimental Diets	Diet Price (USD/kg)	ECR (USD/kg) ^a^	EPI (USD/Fish) ^b^
Con	1.63	1.43 ± 0.008 ^bc^	0.97 ± 0.008 ^ab^
CBM25J	1.59	1.40 ± 0.037 ^c^	1.02 ± 0.013 ^a^
CBM50J	1.42	1.39 ± 0.043 ^c^	0.90 ± 0.032 ^c^
MM25J	1.60	1.57 ± 0.063 ^ab^	0.96 ± 0.019 ^abc^
MM50J	1.44	1.63 ± 0.087 ^a^	0.81 ± 0.016 ^d^
TBM25J	1.64	1.50 ± 0.014 ^abc^	0.98 ± 0.009 ^ab^
TBM50J	1.52	1.42 ± 0.029 ^bc^	0.92 ± 0.023 ^bc^
*p*-value		*p* < 0.02	*p* < 0.0001
Main effect: substitution source			
CBM		1.39 ± 0.025 ^B^	0.96 ± 0.030 ^A^
MM		1.60 ± 0.049 ^A^	0.88 ± 0.035 ^B^
TBM		1.46 ± 0.023 ^B^	0.95 ± 0.018 ^A^
Main effect: substitution level			
25%		1.49 ± 0.041	0.99 ± 0.014 ^A^
50%		1.48 ± 0.058	0.88 ± 0.026 ^B^
Two-way ANOVA			
Substitution source		*p* < 0.004	*p* < 0.006
Substitution level		*p* > 0.7	*p* < 0.0001
Interaction		*p* > 0.4	*p* > 0.1

Con: 55% fish meal (FM)-based diet; CBM25J: dietary 25% FM replacement with chicken by-product meal (CBM) with 22% jack mackerel meal (JMM) inclusion; CBM50J: dietary 50% FM replacement with CBM with 22% JMM inclusion; MM25J: dietary 25% FM replacement with meat meal (MM) with 22% JMM inclusion; MM50J: dietary 50% FM replacement with MM with 22% JMM inclusion; TBM25J: dietary 25% FM replacement with tuna by-product meal (TBM) with 22% JMM inclusion; TBM50J: dietary 50% FM replacement with TBM with 22% JMM; CBM: the CBM-substituted diets; MM: the MM-substituted diets; TBM: the TBM-substituted diets; 25%: dietary 25% FM substitutions; 50%: dietary 50% FM substitutions. Values (means of triplicate ± SE) with different lowercase and uppercase letters indicate statistical differences (*p* < 0.05) based on Duncan’s multiple range test and two-way ANOVA analysis, respectively. ^a^ Economic conversion ratio (ECR, USD/kg) = feed consumption of fish (kg/fish)/weight gain (kg/fish) × diet price (USD/kg). ^b^ Economic profit index (EPI, USD/fish) = [final weight (kg/fish) × fish sale price (USD/kg)] − [ECR (USD/kg) × weight gain (kg/fish)].

## Data Availability

Data are available upon request from the authors.
